# Generative models as computational assays for psychiatry

**DOI:** 10.1192/j.eurpsy.2023.182

**Published:** 2023-07-19

**Authors:** S. Frässle

**Affiliations:** Translational Neuromodeling Unit (TNU), Institute for Biomedical Engineering, University of Zurich & ETH Zurich, Zurich, Switzerland

## Abstract

**Abstract:**

Psychiatry faces fundamental challenges with regard to mechanistically guided differential diagnosis, as well as prediction of clinical trajectories and treatment response. This has motivated novel approaches that aim to develop “computational assays” for inferring patient-specific disease processes from neuroimaging data, which can then be incorporated into decision making in everyday clinical practice. Such computational assays are often based on generative models, which describe how measured data may be caused by a particular mechanism. Combining generative models with machine learning allows translating the inferences from computational assays into patient-specific predictions, an approach referred to as generative embedding.

Here, I illustrate the clinical potential of generative embedding for the exemplary case of a generative model of whole-brain effective (directed) connectivity: *regression DCM* (rDCM). First, I introduce rDCM to the audience and highlight its relevance for understanding the pathophysiology of psychiatric disorders. I then provide an initial demonstration of the clinical utility of rDCM. Specifically, we assessed the ability of rDCM for predicting future episodes of depression in never-depressed adults, using a large dataset (N=906) of resting-state fMRI data from the UK Biobank. Over a 3-year period, half of the participants showed indications of at least one depressive episode, while the other half did not. Using nested cross-validation for training and a held-out test set (80/20 split), we found that a generative embedding procedure based on rDCM in combination with a support vector machine enables statistically significant predictions of future depressive episodes, both on the training (accuracy: 0.63, area under the curve (AUC): 0.66, *p*<0.001) and test set (accuracy: 0.62, AUC: 0.64, *p*<0.001). Interpreting model predictions based on SHAP (Shapley Additive exPlanations) values suggested that the most predictive connections were widely distributed and not confined to specific networks.

In summary, generative models of brain connectivity in general, and rDCM in particular, show initial promise to serve as computational assays for psychiatry. Our analyses suggest that (i) fMRI-based generative embedding approaches have some capacity for early detection of individuals at-risk for depression and (ii) achieving accuracies of clinical utility may require combination of fMRI with other data modalities.

**Disclosure of Interest:**

None Declared

